# High Enantioselective Novozym 435-Catalyzed Esterification of (*R*,*S*)-Flurbiprofen Monitored with a Chiral Stationary Phase

**DOI:** 10.1007/s12010-014-1455-4

**Published:** 2015-01-06

**Authors:** Tomasz Siódmiak, Debby Mangelings, Yvan Vander Heyden, Marta Ziegler-Borowska, Michał Piotr Marszałł

**Affiliations:** 1 Department of Medicinal Chemistry, Collegium Medicum in Bydgoszcz, Nicolaus Copernicus University, Dr. A. Jurasza 2, 85-089 Bydgoszcz, Poland; 2 Department of Analytical Chemistry and Pharmaceutical Technology, Center for Pharmaceutical Research (CePhaR), Vrije Universiteit Brussel-VUB, Laarbeeklaan 103, B-1090 Brussels, Belgium; 3 Department of Chemistry, Chair of Chemistry and Photochemistry of Polymers, Nicolaus Copernicus University, Gagarina 7, 87-100 Toruń, Poland

**Keywords:** Chiral HPLC, Enantioselective esterification optimization, (*R*,*S*)-flurbiprofen, Kinetic resolution, Novozym 435

## Abstract

Lipases form *Candida rugosa* and *Candida antarctica* were tested for their application in the enzymatic kinetic resolution of (*R*,*S*)-flurbiprofen by enantioselective esterification. Successful chromatographic separation with well-resolved peaks of (*R*)- and (*S*)-flurbiprofen and their esters was achieved in one run on chiral stationary phases by high-performance liquid chromatography (HPLC). In this study screening of enzymes was performed, and Novozym 435 was selected as an optimal catalyst for obtaining products with high enantiopurity. Additionally, the influence of organic solvents (dichloromethane, dichloroethane, dichloropropane, and methyl *tert*-butyl ether), primary alcohols (methanol, ethanol, *n*-propanol, and *n*-butanol), reaction time, and temperature on the enantiomeric ratio and conversion was tested. The high values of enantiomeric ratio (*E* in the range of 51.3–90.5) of the esterification of (*R*,*S*)-flurbiprofen were obtained for all tested alcohols using Novozym 435, which have a great significance in the field of biotechnological synthesis of drugs. The optimal temperature range for the performed reactions was from 37 to 45 °C. As a result of the optimization, (*R*)-flurbiprofen methyl ester was obtained with a high optical purity, ee_p_ = 96.3 %, after 96 h of incubation. The enantiomeric ratio of the reaction was *E* = 90.5 and conversion was *C* = 35.7 %.

**Graphical Abstract:**
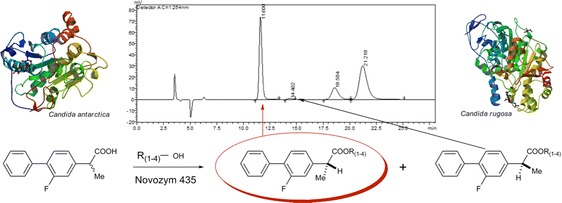
ᅟ

ᅟ

## Introduction

Flurbiprofen [2-(2-fluoro-4-biphenyl)-propionic acid] is a nonsteroidal anti-inflammatory drug of the 2-arylpropionic acid class and has a chiral center. It exists in two enantiomeric forms: (*R*,*S*)-flurbiprofen is currently administered as a racemic mixture, but the enantiomers of this anti-inflammatory agent are characterized by different pharmacological activities [1–3]. (*S*)-flurbiprofen is responsible for the inhibition of the activity of cyclooxygenase Cox-2 as an anti-inflammatory effect and also the inhibition of Cox-1, which causes gastrointestinal side effects after long-term use. In contrast, (*R*)-flurbiprofen is inactive on both forms of cyclooxygenase and is in vivo inefficiently converted into the (*S*)-form. Therefore, it might not cause the gastrointestinal and renal side effects which are characteristic for other NSAIDs [4–6]. Recent studies show that (*R*)-flurbiprofen has antinociceptive properties, inhibits in vivo the progression of colon and prostate cancer in various animal models, and also inhibits in vitro the survival of colon and prostate cancer cell lines. Important is that the inhibition of the Cox enzymes is not considered as the pharmacological mechanism of the activity of the (*R*)-enantiomer. Furthermore, it has been shown that this enantiomer has a significant antiproliferative activity [7–11].

Lipases are the most widely used class of enzymes in chemistry and biotechnology. Presently, these enzymes are useful in organic synthesis and kinetic resolution of racemic compounds. The lipase from *Candida rugosa* and the isoform B of the lipase from *Candida antarctica* are commonly used members of the lipase-group enzymes [12–18]. The active center of the lipase builds a specific environment that allows distinguishing between the enantiomers. Different studies have proven that lipases perform at the interface between hydrophobic and hydrophilic regions, and the water content is an important factor affecting their enantioselectivity. A small amount of water is needed to retain their active three-dimensional conformation state, stability, and active site polarity. Moreover, it should be considered that lipases “tolerate” a large number of non-natural substrates, are stable and active in organic solvents without co-factors, and are commercially available in free and immobilized forms [19–31].

Novozym 435, an immobilized lipase B from *C. antarctica* (CAL-B), is produced by submerged fermentation on a genetically modified *Aspergillus* microorganism and is immobilized by physical adsorption on the macroporous resin Lewatit VP OC 1600; a poly(metacrylic acid) cross-linked with divinylbenzene (DVB). The loading of enzyme (CAL-B) onto the support ranges between 8.5 and 20 % (*w*/*w*). Novozym 435 has an outstanding activity and stability in hydrophobic organic media. However, due to enzyme displacement or leaching, multiple reuse cycles of Novozym 435 are often unfeasible. From a pharmaceutical point of view, well-documented high catalytic activity of Novozym 435 is important, because of the possibility to use this enzyme for the synthesis of selected isomer of a chiral drug [32–38].

The separation of enantiomers and determination of enantiomeric purity of chiral compounds are a significant issue in chemical and pharmaceutical analysis. Among the techniques used for chiral separations, such as gas chromatography (GC), supercritical fluid chromatography (SFC), capillary electrophoresis (CE), and capillary electrochromatography (CEC), the most popular is high-performance liquid chromatography (HPLC) with chiral stationary phases (CSPs). This technique allows separating and determining the enantiomeric purity of many chiral compounds in a simple, efficient, and convenient way. To date, chiral stationary phases based on derivatized polysaccharides, such as cellulose- and amylose derivatives, are the most widely used in liquid chromatography because of their broad enantioselectivity. As mentioned in many reports, cellulose tris(3,5-dimethylphenylcarbamate), amylose tris(3,5-dimethylphenylcarbamate), and cellulose tris(4-methylbenzoate) stationary phases have the ability to chirally resolve more than 80 % of the drugs currently available on the market [39–43].

In the present work, the lipase-catalyzed kinetic resolution of (*R*,*S*)-flurbiprofen has been studied. The optimization of the enzymatic enantioselective esterification was evaluated from the separation with chiral stationary phases. The activity of lipases from *C. rugosa* and *C. antarctica* as potential enzymatic catalysts in the enantioselective esterification of (*R*,*S*)-flurbiprofen was assessed. Additionally, the influences of temperature, solvents as a reaction medium, alcohol moiety, and reaction time were tested on the conversion and enantiomeric ratio of the lipase-catalyzed reactions. The obtained chiral products were analyzed on the chiral stationary phases. The optimization of the chromatographic conditions, such as the selection of stationary and mobile phases, flow rate, temperature, and volume of the injected analytes, allowed obtaining well-resolved peaks of both substrates and products during one chromatographic analysis. The proposed chromatographic separation of (*R*)- and (*S*)-flurbiprofen and their esters within one run was performed in the normal phase conditions on the polysaccharide-based chiral stationary phase—Lux Cellulose-3.

## Materials and Methods

### Chemicals

(*R*,*S*)-Flurbiprofen, (*R*)-flurbiprofen, *n*-heptane, 2-propanol (IPA), *C. antarctica* lipase B (Novozym 435), lipase B *C. antarctica* (recombinant from *Aspergillus oryzae*, powder, beige, ~9 U/mg, CAL-B), lipase CRL type VII from *C. rugosa* (activity ≥700 units/mg solid), and trifluoroacetic acid were purchased from Sigma-Aldrich (Steinheim, Germany). Lipases OF and MY from *C. rugosa* (activity 380,000 and 32,000 U/g solid, respectively) were gifts from Meito Sangyo (Nagoya, Japan). Methyl *tert*-butyl ether (MtBE), dichloromethane (DCM), dichloroethane (DCE), dichloropropane (DCP), methanol, ethanol, *n*-propanol, *n*-butanol, and molecular sieves 4 Å were purchased from POCH (Gliwice, Poland). The (*R*)- and (*S*)-esters of flurbiprofen were obtained as products of a standard esterification reaction of (*R*,*S*)-flurbiprofen and (*R*)-flurbiprofen with appropriate alcohols (methanol, ethanol, *n*-propanol, and *n*-butanol) using sulfuric acid (H_2_SO_4_) as catalyst [44]. The water used in the study was prepared with a Milli-Q Water Purification System (Millipore, Bedford, MA, USA). All incubations were performed at adjusted temperatures (20, 30, 37, and 45 °C) and a fixed number of rotations (600 rpm) in Thermomixer comfort (Eppendorf, Hamburg, Germany).

### Instrumentation

The Shimadzu HPLC system (Japan) used was equipped with a pump, model LC-20AD; a UV-VIS detector, model SPD-20A; a degasser, model DGU-20A_5_; an autosampler, model SIL-20AC_HT_; and a column oven, model CTO-10AS_VP_. A Lux Cellulose-1 (LC-1) (4.6 mm × 250 mm ) column with cellulose tris(3,5-dimethylphenylcarbamate) as chiral selector, a Lux Cellulose-2 (LC-2) (4.6 mm × 250 mm) column with cellulose tris(3-chloro-4-methylphenylcarbamate) as chiral selector, a Lux Cellulose-3 (LC-3) (4.6 mm × 250 mm) column with cellulose tris(4-methylbenzoate) as chiral selector, a Lux Amylose-2 (LA-2) (4.6 mm × 250 mm) column with amylose tris(5-chloro-2-methylphenylcarbamate) as chiral selector, and a Guard Cartridge System model KJO-4282 were purchased from Phenomenex (Torrance, CA, USA). All columns had 5 μm particle sizes.

### Chromatographic Conditions

The most appropriate chromatographic conditions for separation of (*R*)- and (*S*)-flurbiprofen and their esters were a mobile phase composed of *n*-heptane/2-propanol/trifluoroacetic acid (96.5/3.5/0.2 *v*/*v*/*v*) at a flow rate of 1 mL/min. Four types of chiral stationary phases were tested, Lux Cellulose-1, Lux Cellulose-2, Lux Cellulose-3, and Lux Amylose-2, with respect to the resulting peak shapes and the chiral resolutions. The Lux Cellulose-3 HPLC column was chosen as the optimal one for the separation of (*R*)- and (*S*)-flurbiprofen and their esters. The chromatographic process was operated at 15 °C. The UV detection wavelength was set at 254 nm. The enantiomeric excesses of the substrate (ee_s_) and the product (ee_p_) as well as the conversion (*C*), enantiomeric ratio (*E*) (also called enantioselectivity), and resolution values (Rs) were calculated using the equations described in the literature [45–47].

The *E* was calculated as follows:

1 $$ E=\frac{\mathrm{In}\left[\left(1-c\right)\left(1-{\mathrm{ee}}_{\mathrm{s}}\right)\right]}{\mathrm{In}\left[\left(1-c\right)\left(1+{\mathrm{ee}}_{\mathrm{s}}\right)\right]} $$

The ee_s_ and ee_p_ values were determined as follows:

2 $$ {\mathrm{ee}}_{\mathrm{s}}=\frac{R-S}{R+S} $$

3 $$ {\mathrm{ee}}_{\mathrm{p}}=\frac{R-S}{R+S} $$

For *R* > *S*

where *S* and *R* represent the chromatographic peak areas of the *S*- and *R*-enantiomers, respectively. The quantities of flurbiprofen and its esters were expressed by the value of the chromatographic peak areas.

The result values (ee_s_ and ee_p_) were expressed in a percentage using the following equations:

4 $$ \%{\mathrm{ee}}_{\mathrm{s}}=\frac{R-S}{R+S}\times 100 $$

5 $$ \%{\mathrm{ee}}_{\mathrm{p}}=\frac{R-S}{R+S}\times 100 $$

The *C* is

6 $$ c=\frac{{\mathrm{ee}}_{\mathrm{s}}}{{\mathrm{ee}}_{\mathrm{s}}+{\mathrm{ee}}_{\mathrm{p}}} $$

The resolution values (Rs) is

7 $$ \mathrm{R}\mathrm{s}=\frac{2\left({\mathrm{tr}}_2-{\mathrm{tr}}_1\right)}{\left({w}_1+{w}_2\right)} $$

where tr_1_ and tr_2_ are the retention times (in minutes) of the first and the last eluting peaks of a pair, respectively; *w*_1_ and *w*_2_ are baseline widths (in minute) of these peaks.

### (*R*,*S*)-Flurbiprofen Esterification

The reaction mixture was composed of one of the solvent: MtBE, DCM, DCE, or DCP (700 μL), and racemic flurbiprofen (4.8 mg, 0.02 mM) and one of the alcohols: methanol (2.44 μL, 0.06 mM), ethanol (3.52 μL, 0.06 mM), *n*-propanol (4.51 μL, 0.06 mM), and *n*-butanol (5.52 μL, 0.06 mM) as an acyl acceptor, and molecular sieve 4 Å (screening of lipases was performed without addition of molecular sieves). The reaction was started by adding 8.75 mg lipase to the solution (1.5 mL glass tube). The suspensions were incubated at different temperatures (20, 30, 37, and 45 °C) and shaken (600 rpm) in a thermomixer. Samples (50 μL) were withdrawn at several time intervals. The collected samples were dried by evaporation at room temperature and the residues redissolved in 0.9 mL IPA and, after filtration (0.45 μm), injected (5 μL) on the HPLC column.

## Results and Discussion

### Analysis of (*R*,*S*)-Flurbiprofen and its Esters on Chiral Stationary Phases

**Fig. 1:**
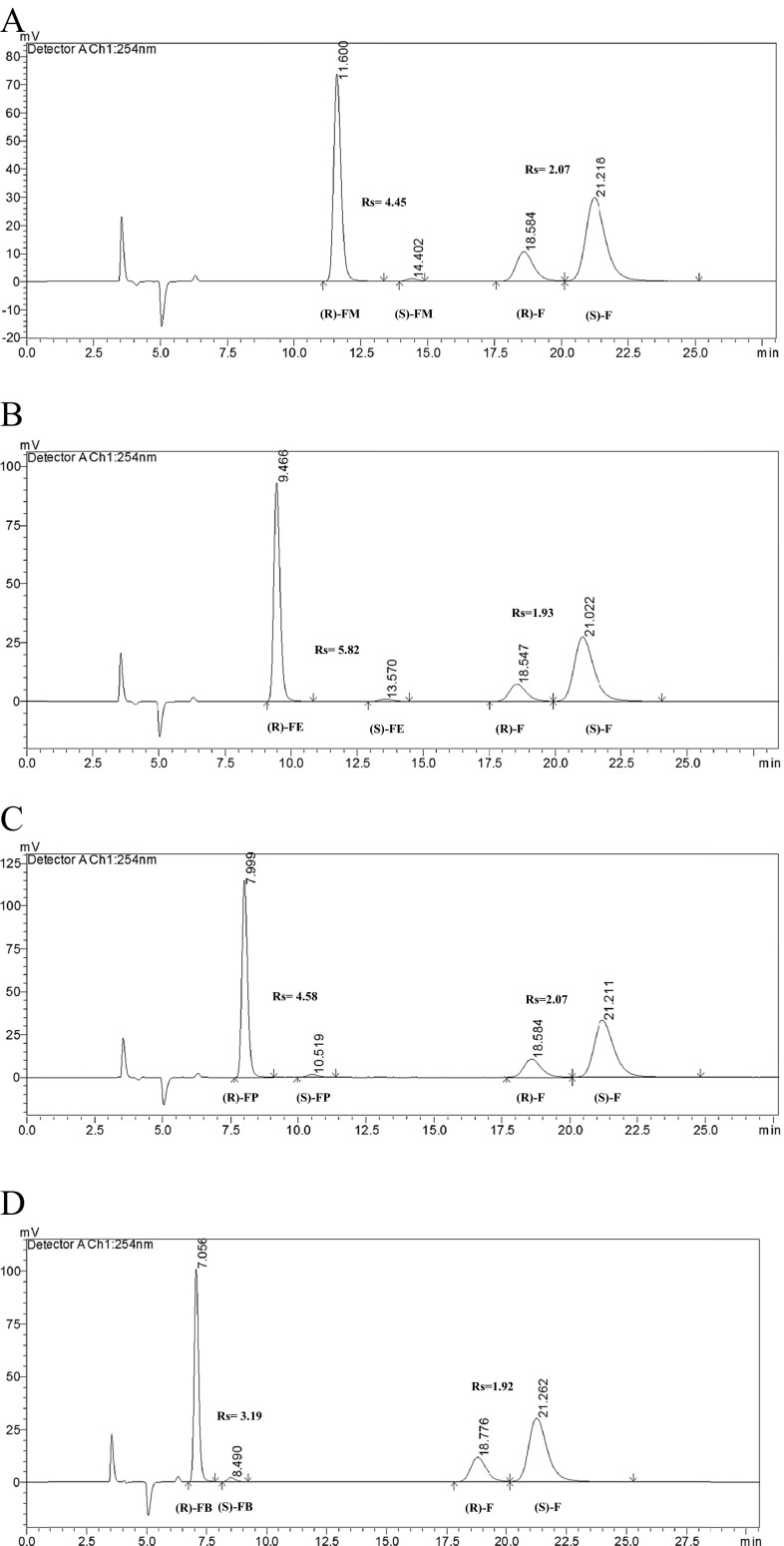
HPLC chromatograms of (*R*,*S*)-flurbiprofen and its esters: **a** (*R*,*S*)-flurbiprofen and its methyl ester, reaction time 96 h; **b** (*R*,*S*)-flurbiprofen and its ethyl ester, reaction time 120 h; **c** (*R*,*S*)-flurbiprofen and its *n*-propyl ester, reaction time 96 h; **d** (*R*,*S*)-flurbiprofen and its *n*-butyl ester, reaction time 48 h; (*R*)-*FM R*-enantiomer of methyl ester, (*S*)-*FM S*-enantiomer of methyl ester, (*R*)-*FE R*-enantiomer of ethyl ester, (*S*)-*FE S*-enantiomer of ethyl ester, (*R*)-*FP R*-enantiomer of *n*-propyl ester, (*S*)-*FP S*-enantiomer of *n*-propyl ester, (*R*)-*FB R*-enantiomer of *n*-butyl ester, (*S*)-*FB S*-enantiomer of *n*-butyl ester, (*R*)-*F R*-flurbiprofen, (*S*)-*F S*-flurbiprofen. Chromatographic conditions: Lux Cellulose-3 (4.6 mm × 250 mm × 5 μm) column; mobile phase: *n*-heptane/2-propanol/trifluoroacetic acid (96.5/3.5/0.2 *v*/*v*/*v*), flow rate = 1 mL/min, *t* = 15 °C, UV = 254 nm

Using the previously proposed methodology by Matthijs et al. [41] and optimization of the chromatographic parameters on the four tested commercial polysaccharide-based CSPs, the LC-3 column in normal-phase mode was selected for the enantioselective separation of (*R*,*S*)-flurbiprofen and its esters (Fig. 1). The main aim of the optimization strategy was to obtain an acceptable baseline resolution (Rs > 1.5) of the compounds, analysis time, and peak shape. Only LC-3 provided acceptable parameters of enantioseparation. The other three columns (LC-1, LC-2, and LA-2) demonstrated a lower (Rs < 1.5) or no (Rs = 0) ability to resolve (*R*,*S*)-flurbiprofen and its esters, long elution times, and inappropriate peak shapes. The optimized mobile phase for LC-3 was composed of *n*-heptane/2-propanol/ trifluoroacetic acid (96.5/3.5/0.2 *v*/*v*/*v*) at a flow rate of 1 mL/min. The normal-phase liquid chromatography (NPLC) analyses were performed at a temperature of 15 °C. The tested compounds ((*R*,*S*)-flurbiprofen and its esters) were eluted within 24 min and showed appropriate peak shapes and baseline resolution (Rs > 1.5).

### Screening of Lipases

**Fig. 2:**
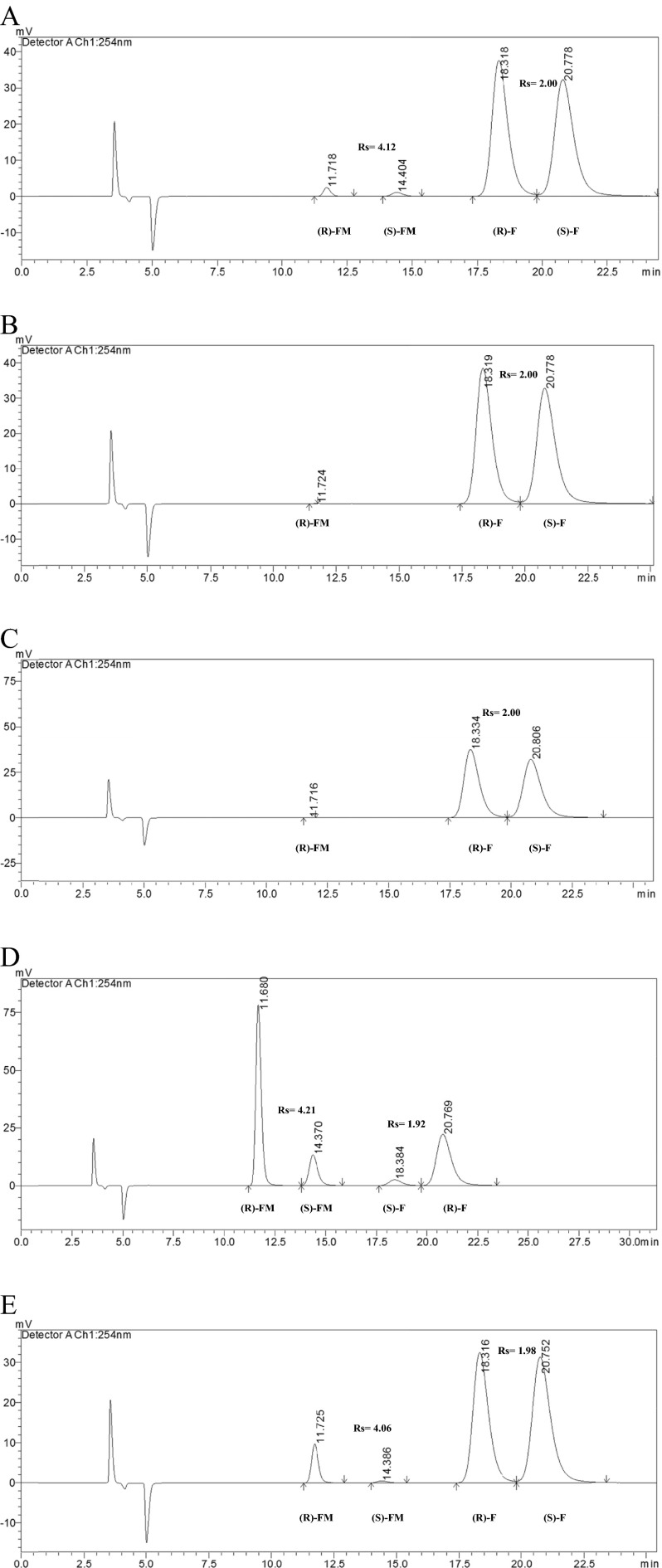
HPLC chromatograms of (*R*,*S*)-flurbiprofen and its methyl esters after 24 h of the reaction: **a** with the use of lipase from *Candida rugosa* OF; **b** with the use of lipase from *C. rugosa* MY; **c** with the use of lipase from *C. rugosa* CRL; **d** with the use of lipase from *Candida antarctica*-Novozym 435; **e** with the use of lipase from *C. antarctica* CAL-B. (*R*)-*FM R*-enantiomer of methyl ester, (*S*)-*FM S*-enantiomer of methyl ester, (*R*)-*F R*-flurbiprofen, (*S*)-*F S*-flurbiprofen. Chromatographic conditions: Lux Cellulose-3 (4.6 mm × 250 mm × 5 μm) column; mobile phase: *n*-heptane/2-propanol/trifluoroacetic acid (96.5/3.5/0.2 *v*/*v*/*v*), flow rate = 1 mL/min, *t* = 15 °C, UV = 254 nm

Commercially available lipases from *C. rugosa* (OF, MY, CRL) and *C. antarctica* (CAL-B and Novozym 435) were tested for their catalytic properties of the enantioselective esterification of racemic flurbiprofen with methanol as an alcohol in dichloropropane. The performed study demonstrates the ability of several tested lipases to enantioselectively catalyze the esterification of (*R*,*S*)-flurbiprofen (Table 1). Our study demonstrated that in case of *C. antarctica* lipase application, (*R*)-flurbiprofen is the faster reacting enantiomer and is enantioselectively esterified to the (*R*)-ester of flurbiprofen. However, the stereopreference of the lipase from *C. rugosa* could not be proved due to the very low enantiomeric ratio of the esterification of (*R*,*S*)-flurbiprofen catalyzed by this lipase in dichloropropane (Fig. 2).

**Table 1:** Screening of lipases for the enantioselective esterification of (*R*,*S*)-flurbiprofen after (*A*) 24 h and (*B*) 330 h of the reaction

Time (h)	Lipase	ee_s_ (%)	ee_p_ (%)	*C* (%)	*E*
A					
24	CAL-B	6.0	84.9	6.5	13.0
24	Novozym 435	82.3	58.0	58.7	9.2
24	CRL	−	−	−	−
24	MY	−	−	−	−
24	OF	0.8	18.0	4.3	1.5
B					
330	CAL-B	72.5	69.35	51.1	11.8
330	Novozym 435	48.0	2.7	94.6	1.4
330	CRL	−	−	−	−
330	MY	−	−	−	−
330	OF	4.0	26.0	13.25	1.8

Reaction conditions: racemic flurbiprofen (4.8 mg, 0.02 mM), methanol (2.44 μL, 0.06 mM), lipases (8.75 mg), dichloropropane (700 μL), reaction temp 37 °C, shaking at 600 rpm

*C* conversion, *ee*_*s*_ enantiomeric excess of the substrate, *ee*_*p*_ enantiomeric excess of the product, *E* enantiomeric ratio, (−) no reaction

Of all the tested lipases (Table 1), only CAL-B, Novozym 435, and OF showed catalytic activity, whereas a lack of activity for the CRL and MY enzymes was observed. It should be noted that the screening of lipases was performed without addition of molecular sieves. Their application in the reaction catalyzed by lipase in the native (not immobilized) form causes a significant reduction of enantiomeric ratio and conversion (data not shown), which has been proven in our laboratory. Although none of the tested lipases showed enough high enantiomeric ratio (minimum for acceptable resolution is *E* > 20 [46]), Novozym 435 was selected for further research, based on the obtained results of *C*, ee_s_, and ee_p_.

### Selection of the Reaction Medium

It is known that the conformation of enzymes strongly depends on the reaction medium (solvents). Hence, the solvents have a meaningful impact on the activity of biocatalysts and thus on the enantiomeric ratio and conversion of the enzymatic reactions. It is reported in the literature [42] that due to the enzyme activity, enantioselective esterification with the use of lipases should be conducted in hydrophobic solvents. Because of the low solubility of (*R*,*S*)-flurbiprofen in commonly used hydrophobic solvents (e.g. isooctane, cyclohexane), other alternative solvents DCM, DCE, DCP, and MtBE were applied in our study as reaction medium. Results indicate (Table 2) that almost all performed esterification reactions in tested solvents were enantioselective. After 20 h of incubation, the highest values of conversion (*C* = 55.5 %) and enantiomeric excess of substrate (ee_s_ = 93.4 %) were achieved when the reaction was carried out in DCP. However, the value of enantiomeric excess of product (ee_p_) in this medium was not satisfactory (ee_p_ = 74.8 %). The enzymatic activity of Novozym 435 in DCE also was high with enantiomeric ratio of the reaction *E* = 62.7 and enantiomeric excess of the product ee_p_ = 94.7 %. The *E*-value after 330 h of incubation dramatically decreased when DCP and MtBE were used as reaction medium. Also, the enantiomeric ratio of the reaction conducted in DCE declined but remained above 20. Interestingly, the enantiomeric ratio of the reaction performed in DCM for both time intervals was at high level (*E* = 93.8 after 20 h and *E* = 109.1 after 330 h).

**Table 2:** Selection of solvent as reaction medium for kinetic resolution of (*R*,*S*)-flurbiprofen after (*A*) 20 h and (*B*) 330 h of the reaction

Time (h)	Solvent	ee_s_ (%)	ee_p_ (%)	*C* (%)	*E*
A					
20	MtBE	46.6	84.7	35.5	19.2
20	DCP	93.4	74.8	55.5	23.5
20	DCE	52.3	94.7	35.6	62.7
20	DCM	16.7	97.5	14.6	93.8
B					
330	MtBE	48.0	54.6	46.7	5.4
330	DCP	54.2	17.8	75.2	2.3
330	DCE	87.8	79.4	52.5	24.8
330	DCM	77.7	95.7	44.8	109.1

Reaction conditions: racemic flurbiprofen (4.8 mg, 0.02 mM), methanol (2.44 μL, 0.06 mM), Novozym 435 (8.75 mg), solvent (700 μL), molecular sieve 4 Å, reaction temp. 37 °C, shaking at 600 rpm

*C* conversion, *ee*_*s*_ enantiomeric excess of the substrate, *ee*_*p*_ enantiomeric excess of the product, *E* enantiomeric ratio, *MtBE* methyl *tert*-butyl ether, *DCP* dichloropropane, *DCE* dichloroethane, *DCM* dichloromethane

Moreover, the application of molecular sieves in the reaction significantly increased enantiomeric ratio, as can be seen when comparing the results obtained at the stage of lipases screening (Table 1) when Novozym 435 was tested without molecular sieves. The obtained parameters of enzymatic activity lead to the conclusion that the addition of molecular sieves in the reaction medium helps in maintaining a sufficient level of water content and provides proper adsorption of water generated as a by-product during the esterification reaction. Water plays a crucial role in the structure and function of enzymes because of its influence on their active conformation. In addition, an inappropriate amount of water in the reaction medium dramatically decreases the enantiomeric ratio and conversion of the reaction. Finally, because of the promising reaction parameters, DCM was selected as an optimal solvent for further studies on the enantioselective esterification of (*R*,*S*)-flurbiprofen.

### Effect of Alcohol Moiety, Reaction Time, and Temperature

**Scheme 1:**
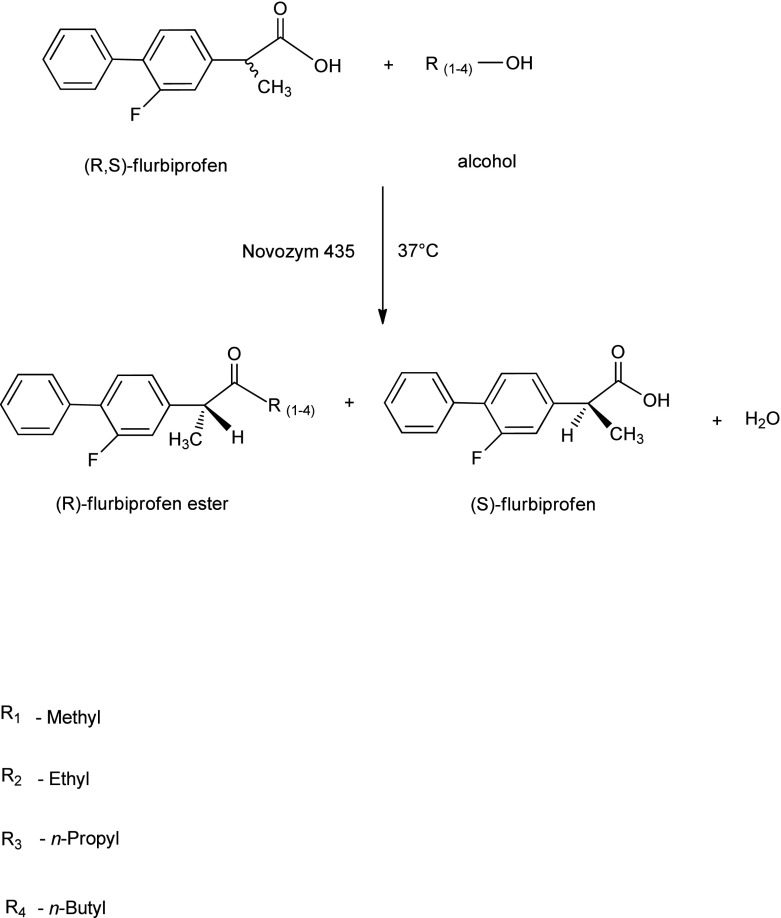
The enantioselective esterification of racemic flurbiprofen with alcohols (methanol, ethanol, *n*-propanol, and *n*-butanol) with the use of Novozym 435

**Fig. 3:**
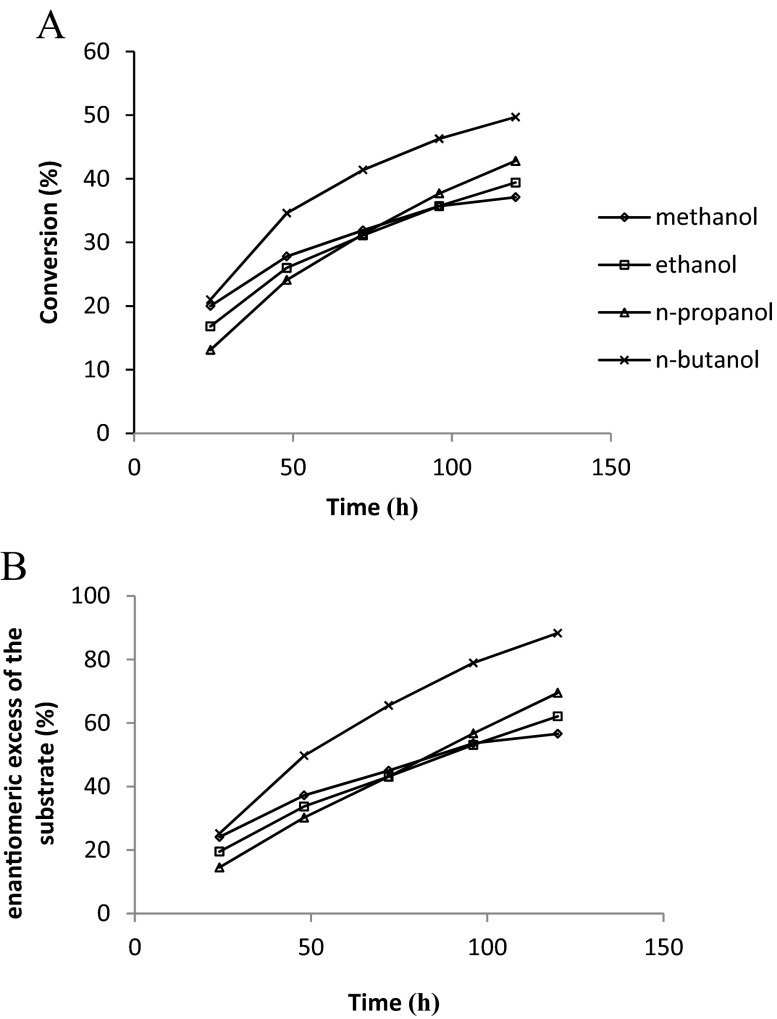
Effect of reaction time **a** on the conversion and **b** on the enantiomeric excess of the substrate. Reaction conditions: racemic flurbiprofen (4.8 mg, 0.02 mM), methanol (2.44 μL, 0.06 mM), ethanol (3.52 μL, 0.06 mM), *n*-propanol (4.51 μL, 0.06 mM), *n*-butanol (5.52 μL, 0.06 mM), Novozym 435 (8.75 mg), dichloromethane (700 μL), molecular sieve 4 Å, reaction temp. 37 °C, shaking at 600 rpm. *C* conversion, *ee*_*s*_ enantiomeric excess of the substrate

A number of studies showed that primary alcohols, because of the steric preferences, are the most effective acyl acceptors in the enzymatic kinetic resolution of chiral acids (Scheme 1) [24, 48]. Hence, the influence of the selected alcohols (methanol, ethanol, *n*-propanol, and *n*-butanol) and the reaction time on the enantiomeric ratio and conversion of the esterification of (*R*,*S*)-flurbiprofen in dichloromethane were tested. It is assumed that the accessibility of the alcohol to the acyl-enzyme intermediate has significant impact on the final reaction yield and enantiomeric ratio. Therefore, the nature of the alcohol moiety and its structure plays an important role in the development of the enantioselective esterification reaction catalyzed by lipase. The comparison of the enzymatic reaction parameters at a similar level of conversion (Table 3) shows that with the increase of the carbon chain length of the studied alcohols, the *E* of the reaction decreases. The best *E*-value (*E* = 90.5) was obtained for methanol, whereas the lowest one was seen when *n*-butanol (*E* = 51.3) was used as an acyl acceptor. Curiously, the esterification reactions of (*R*,*S*)-flurbiprofen catalyzed by Novozym 435 with all used alcohols were significantly enantioselective (*E*-values in the range of 51.3–90.5), which showed the stability of the catalyst in the reaction media with the tested alcohols. Because of the fact that the active site of the enzyme has a hydrophobic character, it is believed that hydrophobic alcohols are the most appropriate for esterification catalyzed by lipase. Furthermore, it is also considered that the shorter polar alcohols, like methanol or ethanol, might cause a low conversion because of their ability to dehydrate and denaturate the enzyme. The results of the conversion reaction after 120 h of incubation were analyzed (37.1, 39.4, 42.8, 49.7 % in methanol, ethanol, *n*-propanol, and *n*-butanol, respectively), and a correlation between the carbon chain length of the alcohol and the final conversion was observed (Table 3, Fig. 3a). These and the previous results allow confirming that with an increased number of carbon atoms in the alcohols, there is an increase in the reaction rate [43].

**Table 3:** Effect of alcohol moiety as an acyl acceptor (*A*) methanol, (*B*) ethanol, (*C*) *n*-propanol, and (*D*) *n*-butanol and reaction time on the parameters of kinetic resolution of (*R*,*S*)-flurbiprofen

Time (h)	ee_s_ (%)	ee_p_ (%)	*C* (%)	*E*
A				
24	24.1	96.7	20.0	77.2
48	37.2	96.6	27.8	84.2
72	45.0	96.1	31.9	77.4
96	53.6	96.3	35.7	90.5
120	56.5	95.6	37.1	79.8
B				
24	19.5	96.2	16.8	63.1
48	33.7	95.8	26.0	65.4
72	43.1	95.7	31.1	69.6
96	53.1	95.5	35.7	74.5
120	62.1	95.3	39.4	78.7
C				
24	14.5	96.5	13.1	65.7
48	30.2	95.2	24.1	54.1
72	43.1	94.4	31.3	53.4
96	56.7	93.9	37.7	56.3
120	69.5	92.3	42.8	55.6
D				
24	25.2	95.4	21.0	53.8
48	49.7	94.0	34.6	53.0
72	65.5	92.6	41.4	51.3
96	78.9	91.4	46.3	53.8
120	88.3	89.4	49.7	52.7

Reaction conditions: racemic flurbiprofen (4.8 mg, 0.02 mM), methanol (2.44 μL, 0.06 mM), ethanol (3.52 μL, 0.06 mM), *n*-propanol (4.51 μL, 0.06 mM), *n*-butanol (5.52 μL, 0.06 mM), Novozym 435 (8.75 mg), dichloromethane (700 μL), molecular sieve 4 Å, reaction temp. 37 °C, shaking at 600 rpm

*C* conversion, *ee*_*s*_ enantiomeric excess of the substrate, *ee*_*p*_ enantiomeric excess of the product, *E* enantiomeric ratio

**Fig. 4:**
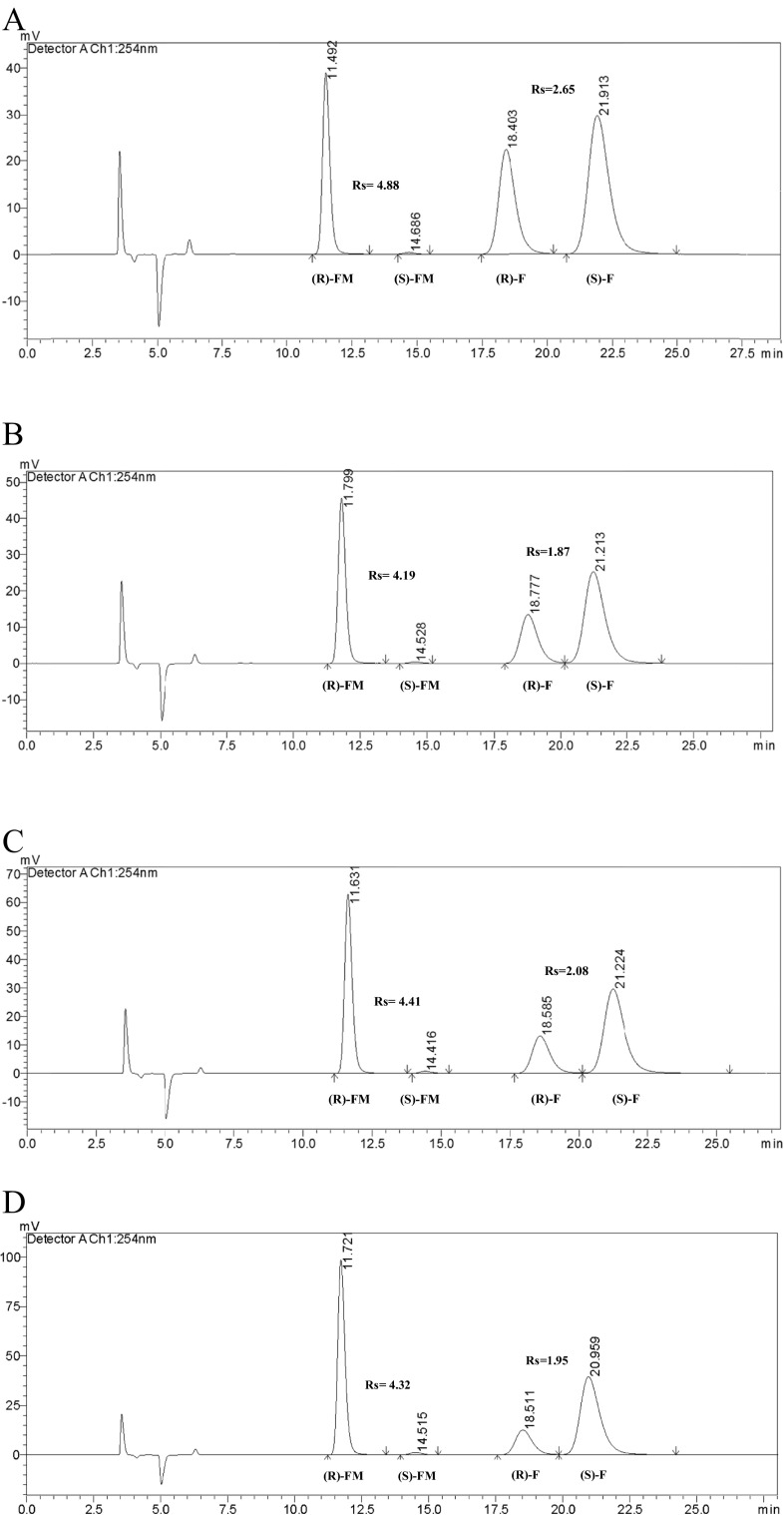
HPLC chromatograms of racemic flurbiprofen and its methyl esters: **a** reaction time 24 h; **b** reaction time 48 h; **c** reaction time 72 h; **d** reaction time 120 h. (*R*)-*FM R*-enantiomer of methyl ester, (*S*)-*FM S*-enantiomer of methyl ester, (*R*)-*F R*-flurbiprofen, (*S*)-*F S*-flurbiprofen. Chromatographic conditions: Lux Cellulose-3 (4.6 mm × 250 mm × 5 μm) column; mobile phase: *n*-heptane/2-propanol/trifluoroacetic acid (96.5/3.5/0.2 *v*/*v*/*v*), flow rate = 1 mL/min, *t* = 15 °C, UV = 254 nm

Regarding the effect of the reaction time on *C*, it is observed that at each interval (24 h) of sampling using methanol, ethanol, and *n*-propanol, *C* was increased at comparable rates, while for *n*-butanol it was higher (Fig. 3a). Likewise, the values of enantiomeric excess of acid (ee_s_) were increased at different rates (Fig. 3b), but with a similar profile as run for C, and thus the highest values were obtained using *n*-butanol. The enantiomeric excess of the product (ee_p_) of esterification when applying methanol (Table 3, Fig. 4) or ethanol as acyl acceptors remained rather constant over time, while for *n*-propanol and *n*-butanol, ee_p_ slightly decreased over time.

The influence of temperature (Table 4) on the conversion and enantiomeric ratio of the esterification of (*R*,*S*)-flurbiprofen was investigated at temperatures ranging from 20 to 45 °C. It is well reported in the literature [48] that temperature has a pronounced effect on the enzyme activity. Due to a better mass transfer and therefore an increased frequency of interactions between substrates and enzyme, a higher temperature significantly contributes to the increased catalytic activity of enzymes. However, an excessively high temperature can cause denaturation of the protein structure [49]. The study showed that the conversion and enantiomeric excess of the substrate (ee_s_) increases with the increase of reaction temperature for all tested alcohols. Comparing these values with a respect to the concerned acyl acceptors, there was approximately almost twofold increase of *C* (23.2 and 40.4 % for methanol, 25.4 and 42.6 % for ethanol, 25.0 and 43.3 % for *n*-propanol, 25.4 and 44.3 % for *n*-butanol) and more than twofold increase of ee_s_ (29.0 and 64.4 % for methanol, 32.3 and 69.2 % for ethanol, 31.3 and 70.3 % for *n*-propanol, 32.1 and 73.2 % for *n*-butanol) between the lowest (20 °C) and the highest (45 °C) temperature, respectively. The highest values of the described parameters were obtained for *n*-butanol (*C* = 44.3 % and ee_s_ = 73.2 %) when the reaction was performed in 45 °C. A slight influence of the temperature was observed on the enantiomeric excess of product (ee_p_), whose values marginally decreased at higher temperature. The application of methanol allowed to obtain the highest value of ee_p_ (96.1 %) in reaction conducted at 37 °C, and the lowest ee_p_ (91.8 %) was achieved when *n*-butanol in 45 °C was used. Summarizing the results, the optimal temperature range for kinetic resolution, based on the conversion, ee_p_, and *E*-value is between 37 and 45 °C.

**Table 4:** Influence of temperature on the parameters of enantioselective esterification of (*R*,*S*)-flurbiprofen with (*A*) methanol, (*B*) ethanol, (*C*) *n*-propanol, and (*D*) *n*-butanol

Temperature (°C)	ee_s_ (%)	ee_p_ (%)	*C* (%)	*E*
A				
20	29.0	95.8	23.2	62.0
30	35.0	96.0	26.7	70.6
37	45.0	96.1	31.9	77.4
45	64.4	95.0	40.4	77.0
B				
20	32.3	94.5	25.4	49.0
30	40.3	94.0	30.0	47.7
37	43.1	95.7	31.1	69.6
45	69.2	93.2	42.6	59.3
C				
20	31.3	94.2	25.0	45.6
30	40.0	94.0	29.8	49.8
37	43.1	94.4	31.3	53.4
45	70.3	92.1	43.3	51.3
D				
20	32.1	94.4	25.4	47.2
30	52.5	94.0	35.8	53.7
37	65.5	92.6	41.4	51.3
45	73.2	91.8	44.3	51.7

Reaction conditions: racemic flurbiprofen (4.8 mg, 0.02 mM), methanol (2.44 μL, 0.06 mM), ethanol (3.52 μL, 0.06 mM), *n*-propanol (4.51 μL, 0.06 mM), *n*-butanol (5.52 μL, 0.06 mM), Novozym 435 (8.75 mg), dichloromethane (700 μL), molecular sieve 4 Å, reaction temperature (20,30,37,45 °C), shaking at 600 rpm, reaction time 72 h

*C* conversion, *ee*_*s*_ enantiomeric excess of the substrate, *ee*_*p*_ enantiomeric excess of the product, *E* enantiomeric ratio

Taking into account the influence of alcohol moiety, reaction time, and temperature on the enantioselective esterification of (*R*,*S*)-flurbiprofen, the global optimal conditions are characterized by temperature in the range of 37–45 °C, application of methanol as an acyl acceptor, and reaction time of 96 h (Fig. 1a).

In the literature, the investigation of Novozym 435- catalyzed kinetic resolution of (*R*,*S*)-flurbiprofen by enantioselective esterification has been described. For example, A. Ghanem [42] studied esterification of (*R*,*S*)-flurbiprofen in dichloromethane and obtained *n*-butyl ester of (*R*)-flurbiprofen with the ee_p_ = 91.3 % after 72 h of incubation. The *E* of the reaction was 25.8, *C* = 14.6 % and ee_s_ = 15.6 %. H.Y. Zhang et al. [50] tested esterification of (*R*,*S*)-flurbiprofen with methanol as acyl acceptor in different solvents and achieved *E* of the reaction in the range of 1.6–7.4. While in small scale-up of the reaction performed in medium composed of dimethylformamide and cyclohexane, the values of ee_s_ = 91 % and *C* = 67 % were received. L. Tamborini et al. [10] obtained the following reaction parameters: ee_s_ = 52 %, ee_p_ = 86 %, *C* = 38 %, and *E* = 22 for the esterification of (*R*,*S*)-flurbiprofen with *n*-butanol after 6 h of incubation in toluene in batch; whereas in a flow-chemistry reactor, after 15 min of the reaction, the authors achieved the following values of the esterification: ee_s_ = 50 %, ee_p_ = 90 %, *C* = 36 % and *E* = 31. R. Morrone et al. [51] carried out the esterification of (*R*,*S*)-flurbiprofen with *n*-propanol and received enantiomeric ratio in the range of 1.0–22.2, depending on the applied reaction medium. However, in a gram scale of esterification with methanol in acetonitrile via a twofold kinetic resolution, (*S*)-flurbiprofen with ee_s_ > 95 % was obtained. All esterification reaction studies published in the above literature examples were performed in different conditions. Comparing the described data with results in our article, it can be seen that the received parameters for all tested alcohols are characterized by high value of enantioselectivity–enantiomeric ratio in the range of 51.3–90.5 (Table 3). (*R*)-flurbiprofen methyl ester as a product of enantioselective esterification was obtained with a high optical purity ee_p_ = 96.3 % after 96 h of incubation. The enantiomeric ratio of the reaction was 90.5, the conversion was 35.7 %, and enantiomeric excess of the substrate, 53.6 %. Despite of reported literature data describing enantioselective esterification of (*R*,*S*)-flurbiprofen with the use of Novozym 435, the detailed optimization of the reaction conditions is still important area of biotechnology, which allows to achieve high enzymatic activity and values of enantiomeric ratio of the reaction.

## Conclusions

In this study the lipases from *C. rugosa* and *C. antarctica* were tested for their application in the enzymatic kinetic resolution of (*R*,*S*)-flurbiprofen by enantioselective esterification. The screening of the enzymes allowed selecting Novozym 435 as the most appropriate biocatalyst for obtaining products with high enantioselectivity.

It should be emphasized that well-resolved peaks of (*R*)- and (*S*)-flurbiprofen and their esters in one chromatographic run on a Lux Cellulose-3 chiral stationary phase in normal phase conditions were achieved. According to data [10, 42, 50, 51], obtained results of kinetic resolution of (*R*,*S*)-flurbiprofen by enantioselective esterification using Novozym 435 for all tested alcohols are characterized by high enantiomeric ratio (*E* in the range of 51.3–90.5). Therefore, the performed enzymatic reactions have a great significance in the field of biotechnological synthesis of drugs. As a result of optimization of the reaction conditions, it was found that the best solvent for kinetic resolution of (*R*,*S*)-flurbiprofen with the use of Novozym 435 was dichloromethane. All esterification reactions with the tested alcohols were enantioselective, but the highest value of enantiomeric ratio was achieved when methanol was applied. The optimal temperature to perform the reactions was in the range from 37 to 45 °C. The proposed conditions were suitable to obtain (*R*)-flurbiprofen methyl ester with a high optical purity ee_p_ = 96.3 % after 96 h of incubation. The enantiomeric ratio of the reaction was *E* = 90.5, conversion *C* = 35.7 %, and enantiomeric excess of substrate ee_s_ = 53.6 %.
